# Decreased gastrointestinal toxicity associated with a novel capecitabine schedule (7 days on and 7 days off): a systematic review

**DOI:** 10.1038/npjbcancer.2016.6

**Published:** 2016-03-30

**Authors:** Karen A Cadoo, Devika Gajria, Emily Suh, Sujata Patil, Maria Theodoulou, Larry Norton, Clifford A Hudis, Tiffany A Traina

**Affiliations:** 1 Breast Medicine Service, Department of Medicine, Memorial Sloan Kettering Cancer Center, New York, NY, USA; 2 Weill Medical College of Cornell University, New York, NY, USA; 3 Department of Epidemiology and Biostatistics, Memorial Sloan Kettering Cancer Center, New York, NY, USA

## Abstract

Capecitabine is widely used in the management of metastatic breast cancer; however, drug delivery is limited by gastrointestinal and other toxicity. We employed mathematical modeling to rationally design an optimized dose and schedule for capecitabine of 2,000 mg twice daily, flat dosing, 7 days on, 7 days off. Preclinical data suggested increased efficacy and tolerability with this novel dosing, and three early-phase clinical trials have suggested a favorable toxicity profile. To further define the tolerability of this regimen, we conducted a systematic review of the gastrointestinal adverse events of patients on these studies. This review demonstrated a favorable gastrointestinal toxicity profile with capecitabine in this novel schedule when given as single agent or in combination therapy with either bevacizumab or lapatinib. No patients discontinued therapy for gastrointestinal toxicity, and there were no grade 4 or 5 gastrointestinal toxicities reported. Grade 3 or greater diarrhea occurred in two (2%); grade 2 or greater mucositis, constipation, and vomiting were reported in three (4%) patients. We conclude that capecitabine administered on a 7 days on, 7 days off schedule has limited gastrointestinal toxicity. Our methodology was based on an analysis of individual patient toxicity data from one phase I single-agent capecitabine and two phase II capecitabine combination studies (with bevacizumab and lapatinib, respectively), focusing specifically on gastrointestinal toxicity.

## Introduction

In spite of significant advances over the last decades in the management of metastatic breast cancer, cure remains an elusive goal. Quality of life on treatment is of major importance and underscores the need to minimize toxicity while preserving or, ideally, improving the efficacy of drugs with known anticancer activity. Models that explore optimum drug delivery are a critical component of rational clinical trial design.^1^

Capecitabine is an orally administered fluoropyrimidine that is absorbed intact through the intestinal wall and converted via three enzymatic reactions to 5-fluorouracil.^2^ The rate-limiting step in this process is the final reaction, catalyzed by thymidine phosphorylase.^2^ In many cancers, including breast and gastrointestinal, thymidine phosphorylase is found in higher levels in tumor than in surrounding normal tissue, allowing relatively selective tumor activity.^2^ Several studies have demonstrated the activity of capecitabine in patients with metastatic breast cancer,^3–5^ and it is widely used in the management of this and gastrointestinal malignancies.^6–8^

The United States Food and Drug Administration (FDA) approved capecitabine at 1,250 mg/m^2^ twice daily, with 14 days continuous dosing followed by a 7-day break (14–7). However, toxicity is a significant issue at this dose and schedule, with the most frequent adverse events being palmar–plantar erythrodysesthesia (PPE), diarrhea, and stomatitis. Diarrhea occurs in 28–54% of patients receiving capecitabine, with grade 3/4 diarrhea experienced by 7–19% of patients.^9^ Dose reductions are required in 41–65% of patients receiving capecitabine,^9^ with a median time to dose reduction of <2 months.^10^ Therefore, in clinical practice an empiric schedule of 1,000 mg/m^2^ twice daily, 14 days on, 7 days off, is frequently employed to improve tolerability.^9,10^ Toxicity is still a significant issue; dose modification is frequently required, and the efficacy of these alternative schedules has not been prospectively determined.^9,11^ Of note, a phase II prospective study failed to demonstrate non-inferiority for an empirically selected lower dose capecitabine regimen.^12^

Norton–Simon modeling can accurately predict future behavior of tumor systems on the basis of early growth measurements.^13^ In addition, it has determined that the rate of tumor regression is proportional to the rate of tumor growth.^1,14^ This model suggests that chemotherapy delivery at increased dose density can increase cancer cell kill by minimizing the opportunity for regrowth between cycles of therapy.^1^ This concept has been borne out in the improved outcomes demonstrated with ‘dose dense’ adjuvant chemotherapy in breast cancer.^15^ Our group applied Norton–Simon modeling to capecitabine dosing and observed that the maximum effect of capecitabine is reached at ~8 days.^16^ Therefore, further dosing through day 14 contributes to toxicity without enhancing efficacy.^16^ This provided a rationale for an alternative, optimized dosing schedule that has been rationally rather than empirically designed.

Preclinical xenograft models testing a 7 day on, 7 day off (7–7) schedule allowed for higher dosing and increased efficacy compared with conventional (14–7) scheduling.^16^ A phase I study of single-agent capecitabine established that the maximum-tolerated dose of capecitabine in this novel schedule is 2,000 mg flat dose twice daily.^17^ Two phase II studies confirmed the feasibility of this capecitabine schedule in combination with the biologic agents lapatinib and bevacizumab for patients with metastatic HER2-positive and HER2-negative breast cancer, respectively.^18,19^ Despite the potentially higher daily dose administered with this regimen, capecitabine was well tolerated in the 7–7 schedule. In particular, we observed improved gastrointestinal tolerability with >grade 2 diarrhea of just 5–26% reported on the individual studies of the 7–7 schedule.^17–19^ To further define this tolerability, we conducted a systematic review of the gastrointestinal toxicity observed in patients on these early-phase studies that explored the capecitabine (7–7) dosing schedule.

## Results

### Patient population

This analysis includes 81 patients evaluable for toxicity; 18 received single-agent capecitabine, 40 received capecitabine and bevacizumab, and 23 received capecitabine and lapatinib. The median age of patients was 52 years (range, 29–73 years) and the median ECOG performance status was 0. Sixty-two patients (76%) had visceral tumor involvement. The median number of prior chemotherapies for metastatic breast cancer was 0 (0–2).

### Therapy delivery

The median number of treatment cycles delivered was 6 (range, 1–40). Seventeen patients (21%) received ⩾12 months of therapy. No patients discontinued therapy for gastrointestinal toxicity. Treatment discontinuation was due to progressive disease in 54 patients (67%), toxicity in 15 (19%), withdrawal of consent in 4 (5%), and other reasons in 8 (10%). Of the patients who discontinued therapy for toxicity, one was receiving single-agent capecitabine, 10 were receiving capecitabine with bevacizumab, and 4 were receiving capecitabine with lapatinib. Treatment discontinuations for toxicity deemed possibly related to capecitabine included palmar–plantar erythrodysesthesia in six patients and laboratory abnormalities in four. Other reasons for patients to discontinue therapy for toxicity included atrial fibrillation, pulmonary embolus, and headache attributed to bevacizumab; rash attributed to lapatinib; and gout. Dose reduction or delay due to gastrointestinal toxicity occurred in five (6%) and four (5%) patients, respectively.

### Gastrointestinal toxicities

These regimens were well tolerated with minimal gastrointestinal toxicity. Table 1 lists the treatment-related gastrointestinal toxicities of all grades. There were no grade 4 or 5 gastrointestinal toxicities reported. Grade 3 or greater diarrhea occurred in two (2%) patients; grade 2 or greater mucositis, constipation, and vomiting were reported in three (4%) patients.

### Palmar plantar erythrodysesthesia (PPE)

There were no grade 4 or 5 PPE events. Grade 3 PPE occurred in 12 (15%) and grade 2 in 32 (40%) patients.

## Discussion

Capecitabine is widely used in the management of metastatic breast and gastrointestinal cancers. Given the palliative goal of therapy for advanced disease in these malignancies, therapeutic tolerance of active agents is of critical importance. Unfortunately, gastrointestinal toxicity is a limiting issue with this drug at the conventional dose and schedule.^9^

Despite the widespread use of this drug, the optimal capecitabine dose for patients with metastatic breast cancer has not been defined.^20^ A number of empirically designed capecitabine dosing schedules have been employed in an effort to increase tolerance and facilitate continued drug delivery.^9,12^ However, the efficacy of these regimens has not been prospectively demonstrated,^9,11^ and it is not clear that this empiric dosing has validity. In fact, as noted above, a randomized phase II study failed to demonstrate the non-inferiority of lower dose capecitabine in combination with docetaxel.^12^ In addition, a two-stage study exploring the feasibility of fixed-dose single-agent capecitabine 3,000 mg per day, in divided doses, failed to meet its first-stage response end point and was closed.^21^ We employed Norton–Simon mathematical modeling to rationally design a novel and optimal dose and schedule of capecitabine.^16^ Modeling suggested an optimized regimen of capecitabine twice daily 7–7. In preclinical models, this improved survival over conventional scheduling. Early-phase clinical studies have established the recommended dose and have demonstrated the feasibility of this approach.^16–19^ Data pertaining to the efficacy of this approach is outstanding and is the subject of an ongoing randomized phase III study led by the Latin American and Caribbean Society of Medical Oncology (SLACOM). This trial is comparing conventional capecitabine scheduling with the 7–7 schedule (NCT02028494) and is powered to detect a progression-free survival benefit with the novel regimen.

In this systematic review, we present individual patient gastrointestinal toxicity from one phase I study of single-agent capecitabine and two phase II studies combined with the biologic agents bevacizumab and lapatinib.^17–19^ These data represent a heterogenous patient population receiving these three therapeutic regimens, each with the novel capecitabine backbone schedule as described. There were no grade 4 gastrointestinal toxicities observed with rare occurrences of grade 3 diarrhea and constipation. In addition, the rates of grade ⩾2 gastrointestinal toxicity were minimal, with only diarrhea reaching above 5% incidence. This appears to compare favorably with historical data from previous randomized studies of capecitabine with bevacizumab and lapatinib^22,23^ (Table 2). Miller *et al.* noted ⩾grade 2 diarrhea in 26% and 28% of patients receiving capecitabine alone and capecitabine with bevacizumab, respectively.^22^ Similarly, Geyer *et al.* reported ⩾grade 2 diarrhea in 25% and 33% of patients receiving capecitabine versus capecitabine and lapatinib, respectively.^23^ In that study, 140 (65%) of patients in the capecitabine group and 181 (79%) of patients in the combination group required a dose reduction for toxicity; however, the toxicities necessitating these reductions were not specified. Notably, no patients in the current systematic review came off study for gastrointestinal toxicity, and 17 patients (21%) were on this cytotoxic therapy for over a year.

Hand-foot syndrome,^9^ or PPE, also significantly limits capecitabine delivery. Exploration of this symptom was not the primary end point of this review; however, given the significance of this toxicity clinically, it has also been highlighted. Six of the patients who discontinued therapy for toxicity that was possibly related to capecitabine did so for PPE. There was no grade 4 PPE, and the rates of grade 2 and 3 PPE (40% and 15%, respectively) are similar to prior data—Miller *et al.* ^22^ reported 36–42% grade 2 and 24–28% grade 3 PPE, while Geyer *et al.* ^23^ reported 26–32% grade 2 and 7–11% grade 3 PPE.

While the efficacy of this approach has to be determined, the improved gastrointestinal tolerability of this novel regimen does not appear to be at the cost of disease control. The median progression-free survival of the phase I study was not reported; however, in the phase II studies it was 8.0 and 9.4 months when combined with bevacizumab and lapatinib, respectively.^18,19^ Although it is not possible to make cross-trial comparisons, this is reasonable in the context of previous data. The median progression-free survival of the phase III studies of capecitabine with bevacizumab and lapatinib was 4.87 months^22^ and 8.4 months,^23^ respectively.

Acknowledging the limitations of this retrospective systematic review of prospectively collected data, our study provides an encouraging signal about the tolerability of this novel dose and schedule. The favorable gastrointestinal toxicity profile demonstrated may facilitate prolonged drug delivery with reasonable quality of life. We propose that this novel dose and schedule of capecitabine optimizes the therapeutic index. As noted, the efficacy of this novel approach is the subject of an ongoing phase III clinical trial.

The potential for this dose and schedule to decrease toxicity and increase drug compliance has wide-reaching public health implications given the broad use of this drug worldwide in breast and gastrointestinal cancers. In addition, it has the potential to reduce drug cost: in this novel schedule, patients receive capecitabine for 14 days out of every 28, as opposed to 14 out of every 21 in conventional scheduling.

Mathematical modeling employed in the design of this regimen allows for dose and schedule determination based on efficacy by defining the point of maximum tumor perturbation.^16^ This moves away from the traditional drug development focus of maximum-tolerated toxicity rather than therapeutic index. The proposed novel capecitabine regimen also moves away from conventional dosing based on body surface area, a widely used practice in drug development that has not been well validated.^24^ This regimen has significant implications for future drug development and will potentially provide proof of principal that rational dose and schedule design, determined by mathematical models, may efficiently optimize activity and reduce toxicity.

We have demonstrated that this novel dose and schedule of capecitabine causes minimal and tolerable gastrointestinal toxicity. We eagerly await the outcome of the ongoing phase III study that will determine the relative efficacy of this approach.

## Materials and methods

In reaching our conclusions from this review, our methodology, based on an analysis of individual patient toxicity data from one phase I single-agent capecitabine and two phase II capecitabine combination studies (with bevacizumab and lapatinib, respectively), focused specifically on gastrointestinal toxicity.

This analysis combines the primary toxicity data from three prospective single-institution studies exploring the feasibility of a novel capecitabine dosing schedule as previously described.^17–19^ These studies included patients with metastatic breast cancer and measurable disease per RECIST criteria. Patients with ECOG performance status ⩽2 and adequate hematologic, hepatic, and renal function were eligible. Each treatment cycle was 28 days, and disease evaluation occurred every 12 weeks with scans assessed by RECIST. For selected eligibility criteria and the design of each study, see the study characteristics table (Table 3). All studies were approved by the Memorial Sloan Kettering Cancer Center Institutional Review Board, and patients were provided written informed consent. Individual patient toxicity data from these studies were prospectively collected and graded using NCI CTCAE version 3 terminology, and attribution assigned. These data were retrospectively extracted by two independent reviewers for the purposes of this systematic review. The primary end point of interest for this review is the incidence and severity of individual gastrointestinal toxicities (nausea, diarrhea, mucositis, constipation, vomiting) with this novel capecitabine schedule. The maximum grade per patient is used as a summary measure. Given the potential for PPE to limit therapy with capecitabine, the incidence and severity of this toxicity has also been highlighted.

## Figures and Tables

**Table 1:** Gastrointestinal toxicity systematic review

N*=81*	*Grade 1*	*Grade 2*	*Grade 3*	*Grade 4*
Nausea	40 (49%)	2 (2%)	—	—
Diarrhea	39 (48%)	6 (7%)	2 (2%)	—
Mucositis	27 (33%)	2 (2%)	1 (1%)	—
Constipation	22 (27%)	3 (4%)	—	—
Vomiting	14 (17%)	3 (4%)	—	—

Maximal Grade, NCI CTCAE version 3. No Grade 5 toxicities observed.

**Table 2:** Toxicity of two phase III studies of capecitabine combined with biologic agents bevacizumab and lapatinib, respectively

*Dose/schedule*	*Capectabine versus capecitabine + bevacizumab (Miller *et al.^22^)								*Capecitabine versus capecitabine + lapatinib (Geyer *et al.^23^)							
	*Capecitabine 2,500 mg/m^2^ divided doses D 1–14* N*=215*				*Capecitabine 2,500 mg/m^2 ^divided doses D 1–14 bevacizumab 15 mg/kg q 3/52* N*=229*				*Capecitabine* N*=152 2,500 mg/m^2^ divided doses D 1–14*				*Capecitabine 2,000 mg/m^2^ divided doses D 1–14 Lapatinib 1,250 mg daily* N*=164*			
	*G1* N *(%)*	*G2* N *(%)*	*G3* N *(%)*	*G4* N *(%)*	*G1* N *(%)*	*G2* N *(%)*	*G3* N *(%)*	*G4* N *(%)*	*G1* N *(%)*	*G2* N *(%)*	*G3* N *(%)*	*G4* N *(%)*	*G1* N *(%)*	*G2* N *(%)*	*G3* N *(%)*	*G4* N *(%)*
Nausea	NR	NR	NR	NR	NR	NR	NR	NR	42 (28)	18 (12)	3 (2)	—	48 (29)	21 (13)	3 (2)	—
Diarrhea	NR	34 (15.8)	23 (10.1)	—	NR	37 (16.2)	27 (11.8)	—	21 (14)	22 (14)	17 (11)	—	44 (27)	33 (20)	19 (12)	2 (1)
Mucositis/stomatitis	NR	11 (5.1)	—	1 (0.5)	NR	16 (7.0)	4 (1.7)	—	12 (8)	5 (3)	1 (<1)	—	17 (10)	7 (4)	—	—
Constipation	NR	11 (5.1)	—	—	NR	14 (6.1)	1 (0.4)	—	13 (9)	3 (2)	1 (<1)	—	14 (9)	2 (1)	—	—
Vomiting	NR	NR	NR	NR	NR	NR	NR	NR	22 (14)	11 (7)	3 (2)	—	30 (18)	10 (6)	3 (2)	—

Abbreviation: NR, not reported.

**Table 3:** Individual study characteristics

	*Study A* ^17^	*Study B* ^19^	*Study C* ^18^
Study phase	Phase I	Phase II	Phase II
Therapy	Capecitabine 2,000 mg orally twice daily, 7 days on 7 days off	Capecitabine 2,000 mg orally twice daily, 7 days on 7 days off Bevacizumab 10 mg/kg intravenously every 2 weeks	Capecitabine 2,000 mg orally twice daily, 7 days on 7 days off Lapatinib 1250 mg orally daily
Selected patient eligibility criteria	Any number of prior therapies No prior fluoropyrimidine therapy for MBC or as adjuvant therapy within 6 months of enrollment Patients with HER2+ disease were eligible if they were not candidates for trastuzumab therapy	Any number of prior therapies No prior fluoropyrimidine therapy for MBC Patients with HER2+ disease were eligible if they were not candidates for trastuzumab therapy	No more than two prior chemotherapy regimens No prior fluoropyrimidine therapy for MBC HER2+ breast cancer with disease progression on prior trastuzumab therapy
Study design	3+3 Dose escalation	Non-randomized open label phase II	Non-randomized open label phase II
Primary end point	MTD of capecitabine in 7/7 schedule	Overall response rate for the combination	Overall response rate for the combination
Safety assessments	Patients were evaluated for toxicity weekly during cycle 1 and every 2 weeks during subsequent cycles Adverse events graded using NCI CTC version 3.0 (Rockville, MD, USA)	Patients were evaluated for toxicity weekly during cycle 1 and every 2 weeks during subsequent cycles Adverse events graded using NCI CTC version 3.0	Patients were evaluated for toxicity every 2 weeks during cycle 1 and once per cycle thereafter Adverse events graded using NCI CTC version 3.0 LVEF was monitored by MUGA scan every 12 weeks.

Abbreviations: LVEF, left ventricular ejection fraction; MBC, metastatic breast cancer; MTD, maximum-tolerated dose; MUGA, multigated acquisition.
